# Protective role of perivascular adipose tissue in the cardiovascular system

**DOI:** 10.3389/fendo.2023.1296778

**Published:** 2023-12-08

**Authors:** Yi Tong, Zheng Zuo, Xinqi Li, Minghua Li, Zhenggui Wang, Xiaoxue Guo, Xishu Wang, Ying Sun, Dongmei Chen, Zhiguo Zhang

**Affiliations:** ^1^ Center for Cardiovascular Medicine, The First Hospital of Jilin University, Changchun, China; ^2^ Center for Cardiovascular Medicine, The Second Hospital of Jilin University, Changchun, China

**Keywords:** perivascular adipose tissue, anti-inflammatory, vasodilatory, anticontractile, hypertension, atherosclerosis

## Abstract

This review provides an overview of the key role played by perivascular adipose tissue (PVAT) in the protection of cardiovascular health. PVAT is a specific type of adipose tissue that wraps around blood vessels and has recently emerged as a critical factor for maintenance of vascular health. Through a profound exploration of existing research, this review sheds light on the intricate structural composition and cellular origins of PVAT, with a particular emphasis on combining its regulatory functions for vascular tone, inflammation, oxidative stress, and endothelial function. The review then delves into the intricate mechanisms by which PVAT exerts its protective effects, including the secretion of diverse adipokines and manipulation of the renin-angiotensin complex. The review further examines the alterations in PVAT function and phenotype observed in several cardiovascular diseases, including atherosclerosis, hypertension, and heart failure. Recognizing the complex interactions of PVAT with the cardiovascular system is critical for pursuing breakthrough therapeutic strategies that can target cardiovascular disease. Therefore, this review aims to augment present understanding of the protective role of PVAT in cardiovascular health, with a special emphasis on elucidating potential mechanisms and paving the way for future research directions in this evolving field.

## 1 Introduction

Cardiovascular disease is a major cause of illness and death worldwide, through its effects on the heart and blood vessels (1, 2). This collective name covers a variety of diseases, including coronary heart disease, heart failure, and stroke. Despite advances in medical treatments, cardiovascular disease continues to pose significant burdens on both healthcare systems and individuals. In general, perivascular adipose tissue (PVAT) encircles nearly all blood vessels other than those supplying the nerves and pulmonary vasculature (3–6). Traditionally, PVAT has been considered an inert connective tissue that provides structural support to these vessels. However, emerging evidence has revealed that PVAT is not a passive bystander but instead has an active role in cardiovascular disease (7, 8). In this review, we initially summarize the recent research findings and advances related to the cellular origin and organization of PVAT, then discuss the anti-inflammatory, vasodilatory, and anti-constrictive actions of PVAT, and finally examine the potential of PVAT as a future therapeutic target for cardiovascular disease.

## 2 Cellular origin and structure of PVAT

### 2.1 Cellular origin

The complex cellular components of PVAT include various cell types, notably adipocytes, fibroblasts, immune cells, and vascular cells (9, 10). Adipocytes have a central role in PVAT as the primary cells responsible for producing and releasing adipokines, cytokines, and other biologically active molecules (11). Regarding the other cell types, fibroblasts facilitate reshaping of the extracellular matrix to maintain the structural integrity of PVAT (12), immune cells (macrophages, T cells) actively participate in the inflammatory and immune responses of PVAT (13, 14), and vascular cells (smooth muscle cells [SMCs], endothelial cells [ECs]) have an underlying involvement in the vascular function of PVAT (15). Research has indicated that PVAT also contains adipocytes derived from mesenchymal precursors known as SM22α^+^ cells, which are commonly found around the aorta and act as an important source of vascular smooth muscle cells (VSMCs) (16). Other studies have demonstrated that adipose-derived stem cells from percutaneous and visceral adipose tissue can differentiate into various types of vascular cells, including ECs and SMCs (12, 17).

Despite the growing interest in PVAT, the origins of its cellular components have not been fully elucidated. PVAT cells are hypothesized to arise from a variety of sources, including resident progenitor cells in adipose tissue and cells migrating from other tissues (15). Research has shown that adipocyte progenitor cells, also known as preadipocytes, can differentiate into mature adipocytes and contribute to the expansion of PVAT (18). Moreover, recent evidence has suggested that cells from the vascular adventitia, such as pericytes and mesenchymal stem cells, can differentiate into PVAT cells under specific conditions (19).

### 2.2 Tissue structure

Blood vessels have three distinct layers: the intima, media, and adventitia layers. The intima layer primarily consists of ECs, while the media layer is predominantly composed of SMCs. The adventitia layer, which contains nerve endings, can be further categorized into two sublayers: the adventitial compacta and adventitial fat (20). The adventitial compacta primarily contains fibroblasts, while the adventitial fat mainly consists of adipocytes. The terms perivascular fat and PVAT are often used interchangeably with adventitial fat (21).

Regarding adipose tissue itself, there are typically three different types: white adipose tissue (WAT), brown adipose tissue (BAT), and beige adipose tissue (BeAT). Similar to other adipose tissue depots, PVAT is a combination of WAT and BAT, with varying proportions depending on the organs involved (22). Development of brown and white adipocytes is associated with distinct lineages, although there is some overlap, and their exact origins remain uncertain. Thus, further investigations are required to fully understand the regulatory mechanisms and developmental origins of these adipocytes.

WAT is primarily composed of white adipocytes that house singular, sizable lipid droplets, and it is mainly situated in the hypodermis and perivisceral region. It specializes in storing and mobilizing fat, and its metabolic pathways modulate the production of proteins and lipids, which can have significant effects on inflammation and insulin sensitivity both locally and systemically (23).

On the contrary, BAT, which is transiently present in the interscapular and mediastinal regions of humans, has distinctive thermogenic properties and functions in maintaining a stable body temperature. Furthermore, brown-like adipocytes, which are rich in lipid droplets and mitochondria, can regulate body temperature by generating calories through lipid metabolism (24). By employing ^18^F-FDG PET-CT imaging, it has been discovered that functional BAT is prevalent in adults and consumes a substantial amount of energy. Therefore, methods that can increase the volume or activity of BAT have potential as treatments for metabolic diseases (25).

A comprehensive examination of human coronary arteries indicated that PVAT expresses certain genes at intermediate levels between WAT and BAT (26). Understanding the origins and developmental pathways of thermogenic PVAT adipocytes in adults is crucial for the development of therapeutic approaches that can enhance BAT accumulation (9). Notably, PVAT in various parts of the body shows similarities to different types of adipose tissue. The phenotype of PVAT is influenced by its location, with thoracic and abdominal PVAT exhibiting distinct characteristics. Specifically, abdominal PVAT has similarities to WAT, while thoracic PVAT shares more traits with BAT (27–29). Consistent with this, human aortic and coronary PVAT showed similarities with BAT in terms of the expression of BeAT (30). Because mitochondria contain large amounts of UCP1 protein, both BAT and BeAT are thermogenic. Unlike WAT, BAT and BeAT have anti-inflammatory properties, and thus we focused on the cellular origin of thoracic aortic PVAT to examine whether it exerts anti-inflammatory effects.

To determine the proteomic similarities, a comprehensive proteomic analysis was performed on PVAT, BAT, and WAT in ApoE^−/−^ mice, and a principal component analysis of the proteomic profiles revealed common protein expression patterns in PVAT and BAT that distinguished them from WAT (16). Furthermore, the proteomic features of PVAT resembled those of BAT (16). Thoracic PVAT was more similar to traditional BAT than to BeAT from a morphologic and structural point of view. Analyses of the global gene expression profiles using DNA microarrays revealed that PVAT had almost identical gene expression profiles to BAT, with Ucp1, Cidea, and other genes uniquely expressed or highly overexpressed in BAT with similar levels of expression (31, 32).

From a functional standpoint, PVAT plays a crucial role in the regulation of intravascular temperature, similar to BAT. In addition, PVAT exhibits thermogenic properties upon exposure to cold temperatures. In SMPG KO model mice with VSMCs that were rendered deficient in the adipogenic transcription factor peroxisome proliferator-activated receptor-γ (PPARγ) using a SM22α-Cre knock-in strategy (33), the absence of PVAT led to impaired thermogenic activity, resulting in decreased temperature and endothelial dysfunction (34). Notably, the SMPG KO mice had no PVAT due to the absence of PPARγ, resulting in a lack of PVAT surrounding vessels like the thoracic and abdominal aorta. This lack of PVAT further contributed to the decrease in intravascular temperature. In mammals, variations in ambient temperature elicit a vascular reaction that involves the functions of ECs and SMCs. A similar physiological mechanism may exist in humans, whereby the intravascular temperature gradient increases in large veins as blood approaches the heart (35), thus highlighting the critical role for PVAT in the maintenance of vascular homeostasis.

**Figure 1:**
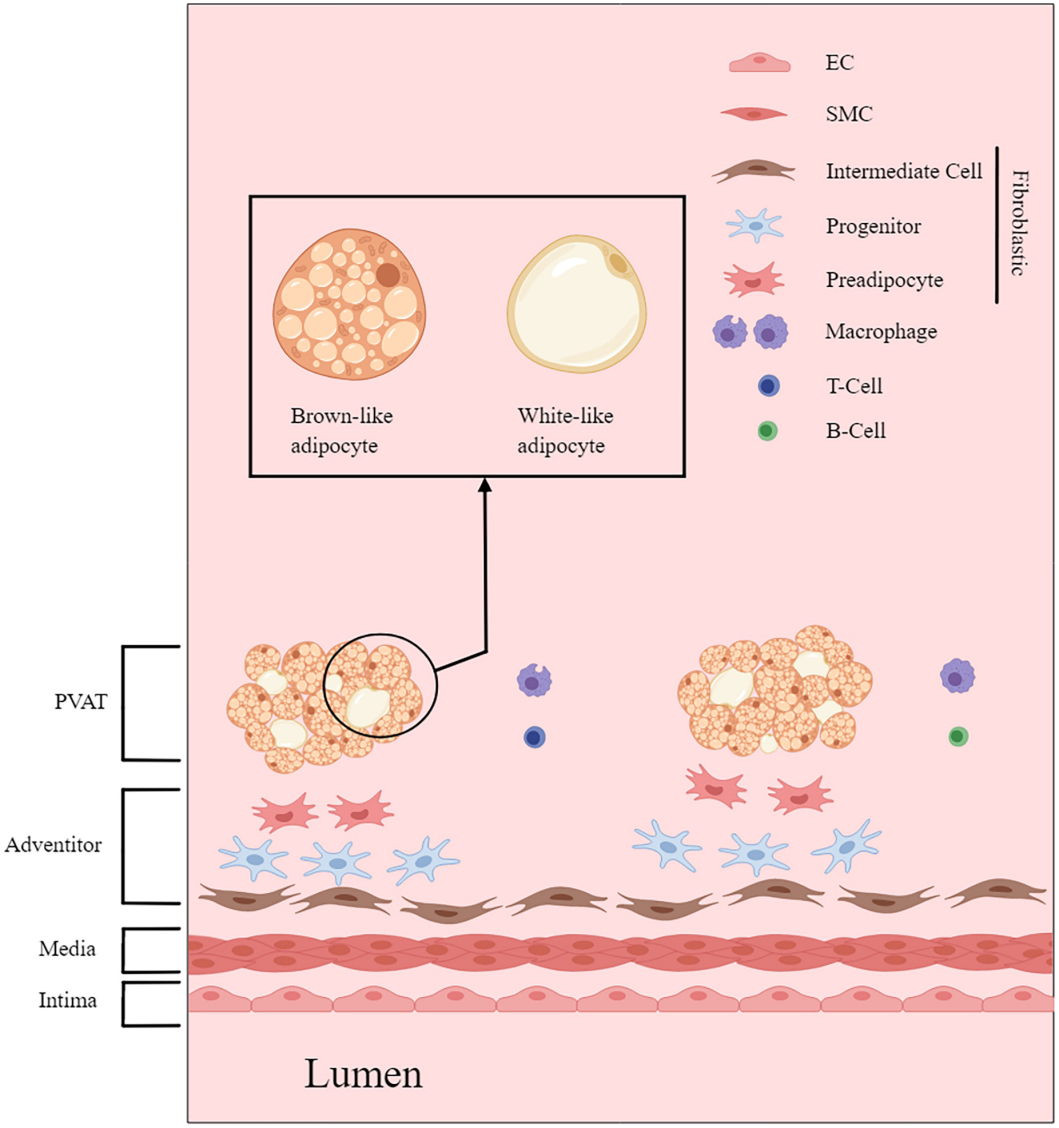
PVAT and the architecture of the vascular wall. This figure is created with MedPeer (www.medpeer.cn).

The structure and functionality of PVAT are also influenced by various physiological parameters, including aging, sex, and race. Mechanical considerations, such as intravascular injuries, also affect the properties of PVAT. In addition to its cellular components, PVAT includes collagens, elastic fibers, nerve fibers, capillaries, and other components (**Figure 1**).

## 3 Vasoprotective effects of PVAT

**Figure 2:**
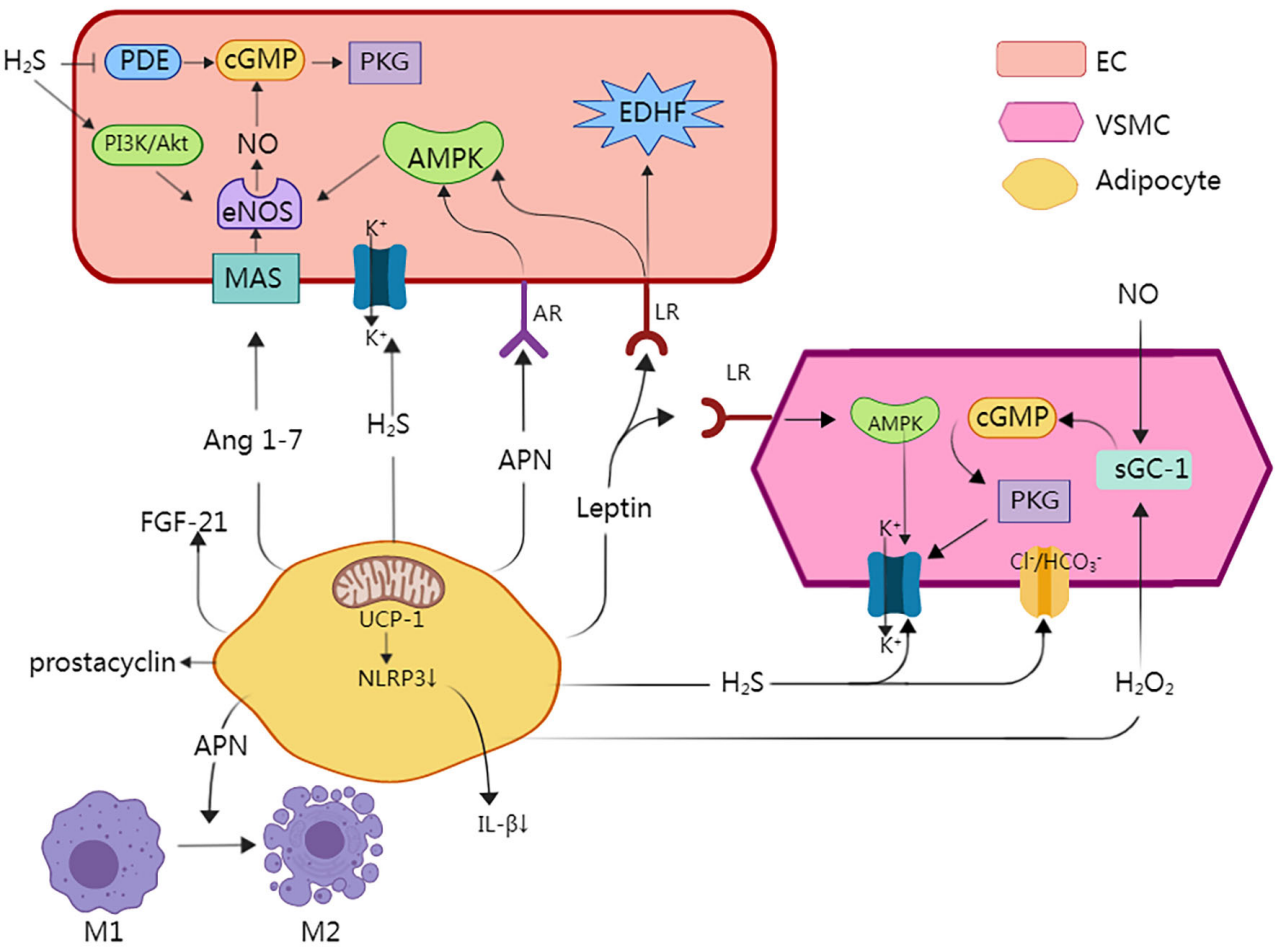
A major underlying mechanism for how PVAT exerts its effects is through the release of substances called adipocyte-derived relaxing factors (ADRFs), which have important roles in modulating vascular structure and function. Adiponectin causes vasodilation by affecting adiponectin receptors (AR) in endothelial cells, which contributes to the activation of locations 5′ adenosine monophosphate-activated protein kinase (AMPK), which is responsible for the activation of endothelial NO synthase (eNOS). Enhanced NO concentration induces activation of cyclic guanosine monophosphate (cGMP), which is responsible for opening large-conductance calcium-activated potassium channels (BKCa). eNOS is present in both endothelial cells and adipocytes. Moreover, Adiponectin directly regulates the phenotype of macrophages and facilitates their transition from the pro-inflammatory M1 macrophages to the anti-inflammatory M2 macrophages. Leptin activates leptin receptors (LR), which are responsible for activation of not only AMPK, but also endothelium-derived hyperpolarizing factor (EDHF), which activates BKCa. Moreover, AMPK independently activates BKCa and induces a hyperpolarization effect. Hydrogen sulfide (H2S) induces activation of BKCa in VSMCs and endothelial cells, and H2S can inhibit the degradation of eNOS and induce its phosphorylation, resulting in NO generation via the PI3K/Akt pathway and p38 MAPK pathway. Moreover, it induces a decrease in intracellular pH by the activation of Cl−/HCO3− ionic exchanger. Angiotensin 1–7 (Ang 1–7) by affecting endothelial Ang 1–7 receptor (MAS) activates eNOS and increases the NO concentration. Prostacyclin interacts with receptors present on blood vessels, and has an important role in vasodilatory properties. Hydrogen peroxide (H2O2) stimulates the soluble guanylyl cyclase (sGC-1), which induces vasodilation through the NO/GC-1/cGMP pathway. UCP1 inhibited the activation of Nod-like receptor family pyrin domain-containing 3(NLRP3) inflammatory and decreases the levels of pro-inflammatory factors such as IL-1β. This figure is created with MedPeer (www.medpeer.cn).

Under physiological conditions, PVAT has a range of beneficial effects, including anti-inflammatory properties, optimization of free fatty acid metabolism, and regulation of vasodilation (36). A major underlying mechanism for how PVAT exerts its effects is through the release of substances called adipocyte-derived relaxing factors (ADRFs), which have important roles in modulating vascular structure and function (7, 11, 37). ADRFs mostly comprise adiponectin, leptin, nitric oxide (NO), hydrogen sulfide (H_2_S), hydrogen peroxide (H_2_O_2_), and fibroblast growth factor-21 (FGF-21), although it was recently found that expression of the mitochondrial inner membrane protein UCP1 and depletion of dendritic cells in PVAT can also increase the anti-inflammatory effects of PVAT (32, 38). The anti-contractile and anti-inflammatory effects of PVAT are summarized in **Tables 1**, **2**; **Figure 2**.

**Table 1:** The anti-contractile and anti-inflammatory effects of PVAT *in vitro* study.

Factor	Role	Function	Reference	Data source
Adiponectin	anti-contractile	Inhibits vascular smooth muscle proliferation *in vivo* and *in vitro* via an AMPK-dependent pathway	(39)	Vitro Study
		Activation of local eNOS function by stimulatory phosphorylation and increased BH_4_ production	(40)	Vitro Study
	anti-inflammatory	Macrophage phenotype favoring the switch from a proinflammatory M1-like state to an anti-inflammatory M2-like state	(41)	Vitro Study
H_2_O_2_	anti-contractile	Stimulates the sGC-1	(42)	Vitro Study
H_2_S	anti-contractile	Opening ATP-sensitive K+ channels reduction in intracellular pH in a dose-related way, further contributing to the vasodilation process	(43, 44)	Vitro Study
		Inhibiting the activity of phosphodiesterase	(45)	Vitro Study
COX-Derived Factors	anti-contractile	Prostacyclin against endothelium dysfunction	(46)	Vitro Study
Angiotensin 1–7	anti-contractile	Inducing vasodilation by endothelium-dependent mechanisms	(47)	Vitro Study
FGF-21	anti-inflammatory	FGF-21 treatment greatly lower IL-6, TNF-α, and MCP-1 expression in adipocytes and stromal vascular fraction (SVF) cells	(48)	Vitro Study

**Table 2:** The anti-contractile and anti-inflammatory effects of PVAT *in vivo* study.

Factor	Role	Function	Reference	Data source
Leptin	anti-contractile	Increase endothelium-dependent vasodilation by AMPK activation	(49)	Vivo Study
		Increase synthesis of NO and endothelium-derived hyperpolarizing factor (EDHF)	(50)	Vivo Study
		Increase norepinephrine turnover in interscapular BAT	(51)	Vivo Study
		Reducing the Ca2+ release from cellular reserves and inducing VSMC proliferation	(49)	Vivo Study
NO	anti-contractile	NO is directly produced and released by eNOS in PVAT	(52)	Vivo Study
UCP1	anti-inflammatory	Blocking mitochondrial superoxide (mtSuperoxide)–induced activation of the NLR family pyrin domain containing 3 (NLRP3) inflammasome and production of interleukin-1β (IL-1β)	(32)	Vivo Study
Dendritic cell depletion	anti-inflammatory	Dendritic cell depletion greatly limit TNF-α and IL-6 generation	(38)	Vivo Study

### 3.1 Anti-contractile effects of PVAT

#### 3.1.1 Adiponectin

PVAT-derived adiponectin is involved in several physiological procedures and has a positive role in vascular homeostasis. In the healthy body under normal conditions, PVAT produces and releases abundant adiponectin (53). This hormone functions as a vasodilator by directly affecting ECs and VSMCs through multiple mechanisms. One way in which adiponectin promotes vasodilation is through endothelial nitric oxide synthase (eNOS) via AMPK-mediated phosphorylation (39). This leads to increased generation of NO, a potent vasodilator. Adiponectin also enhances eNOS function by stimulating the phosphorylation and boosting the generation of BH4, an essential cofactor for eNOS activity (40). Studies in mice lacking AMPKα1, a key regulator of adiponectin signaling, revealed a loss of the vasodilatory effect mediated by PVAT, indicating the importance of AMPK for this process (54). Furthermore, adiponectin was shown to suppress the proliferation of VSMCs both *in vivo* and *in vitro* through an AMPK-related signaling pathway (39). In addition, AMPKα1 knockout mice had significantly lower circulating lipocalin levels, indicating that AMPK is essential for lipocalin generation and lipocalin-mediated vasodilation (54). In summary, PVAT-derived adiponectin exerts beneficial effects on vascular function by enhancing vasodilation and inhibiting VSMC proliferation. The actions of adiponectin are mediated by AMPK-dependent pathways, highlighting the vital role of this signaling molecule in the maintenance of vascular homeostasis.

#### 3.1.2 Leptin

Leptin is an abundant secreted adipokine that has a key role in the regulation of appetite and weight. It is also considered to act as a protective adipokine for cardiovascular function (55). The vasodilation induced by leptin occurs through both endothelium-related and independent methods, with the specific mechanisms depending on the types of blood vessel involved. In major arteries, such as the aorta, leptin enhances endothelium-dependent vasodilation by activating AMPK, a mechanism comparable to that of adiponectin. This activation leads to eNOS phosphorylation and ultimately increased vasodilation. Leptin also targets vascular ECs, impeding the contractile effects of angiotensin II by decreasing calcium release into cellular stores and stimulating VSMC proliferation (49). In smaller arteries, such as the mesenteric artery, leptin triggers an increase in the production of NO and endothelium-derived hyperpolarizing factor (EDHF), both of which contribute to endothelium-related vasodilation (50). Research has demonstrated that leptin acts as an endothelium-dependent vasodilator in coronary artery disease patients, as evidenced by its effects on saphenous and internal mammary artery vascular rings (56). Furthermore, leptin induces vasodilation in human coronary blood vessels and promotes endothelial NO production by ECs in humans (57). While the role of leptin in promoting sympathetic activity is well-established, the exact mechanism by which it counteracts the effects of the sympathetic nerve system on blood pressure remains unclear. One study discovered that leptin induces a hypotensive effect when the effects of the sympathetic nerves are eliminated (56). These hemodynamic effects of leptin coincide with the endothelium-mediated vasodilatory effects induced by the same hormone through NO or EDHF on conduit and resistance arteries, respectively (56).

#### 3.1.3 Nitric oxide

NO is a gas that easily diffuses throughout the body. It is widely recognized to act as a vasodilator, meaning that it can widen blood vessels. NO is synthesized by three different enzymes called eNOS, inducible nitric oxide synthase (iNOS), and neuronal nitric oxide synthase (nNOS) (58). The iNOS enzyme is unique because it does not require calcium ions (Ca^2+^) for its activity and can be stimulated by inflammatory cytokines, indicating its potential involvement in the progression of various inflammatory diseases (59). Meanwhile, the nNOS enzyme is found in the neurons of the central and peripheral nervous systems, and acts as a neurotransmitter for the modulation of blood pressure, and the eNOS enzyme, which is mainly found in ECs, has an anti-atherosclerotic property and functions in local blood pressure control (59). Research has demonstrated that eNOS is expressed not only in ECs but also in PVAT, and that atherosclerosis, a condition characterized by reduced bioavailability of NO, is closely related to endothelial dysfunction (52, 60, 61). In this context, eNOS-derived NO was shown to possess multiple anti-atherosclerotic properties, including the ability to regulate VSMC proliferation and leukocyte adhesion, inhibit platelet aggregation, and reduce vascular inflammation (52, 59). In obese individuals, excess PVAT-derived tumor necrosis factor-α (TNF-α), along with increased expression of endothelin-1(ET-1) and endothelin ETA receptors in blood vessels, disrupts the balance of the ET-1/NO system, leading to impaired release of NO (62). This imbalance is further exacerbated by the overproduction of reactive oxygen species, leading to a loss of coupling with eNOS and reduced NO generation. In contrast, when PVAT was removed in healthy people, basal NO generation in small arteries was decreased, suggesting that PVAT contributes to vascular NO generation (62). NO exerts its vasodilatory effects by relaxing VSMCs through the cGMP-PKG cascade and/or by activating potassium channels in SMCs to induce membrane hyperpolarization (63). In addition, NO can up-regulate the synthesis of H_2_S, another vasodilator, by increasing the availability of its precursor as well as the expression of its synthetic enzyme cystathionine gamma lyase. This interaction between NO and H_2_S further enhances the vasodilatory effects in the body (64).

#### 3.1.4 Hydrogen sulfide

H_2_S is a gas that is synthesized by PVAT, ECs, and VSMCs. Its role in the regulation of vascular tone is crucial. The vasodilation caused by H_2_S is a result of its ability to activate BK channels in VSMCs. This activating effect leads to hyperpolarization of the cytosol, which in turn inactivates voltage-gated L-type Ca^2+^ channels. This cascade of events ultimately leads to a decrease in the intracellular Ca^2+^ concentration (43). Besides its role in vasodilation, H_2_S was shown to reduce intracellular pH in a dose-dependent manner, thus further contributing to the vasodilation process. The underlying mechanism for these effects involves the Cl^−^/HCO^3−^ ion exchanger (65). Another study indicated that there may be an interaction between NO and H_2_S in terms of their production and pathophysiological functions (66). In the cardiovascular and cerebrovascular systems, H_2_S and NO influence one another and rely on each other for the regulation of angiogenesis (67). Research has shown that H_2_S can inhibit the degradation of eNOS and induce its phosphorylation, resulting in NO generation via the PI3K/Akt pathway (68–70) and p38 MAPK pathway (71). H_2_S can also increase cGMP by inhibiting the activity of phosphodiesterase (45). These effects allow H_2_S to exert its important anti-vasoconstrictive actions.

#### 3.1.5 Hydrogen peroxide

H_2_O_2_ produced by PVAT has dual effects on blood vessels, by acting as both a vasoconstrictor and a vasodilator depending on various factors. These factors include the concentration of H_2_O_2_, type of blood vessel, and contractile state of the vessel (72). In healthy individuals, the H_2_O_2_ concentration is typically non-toxic. H_2_O_2_ can permeate the cell membrane and easily diffuse into SMCs, where it stimulates soluble guanylyl cyclase (sGC-1) and acts as a receptor for NO in smooth muscle to induce vasodilation via the NO/sGC-1/cGMP pathway (42). In the obese population, the contractile response to H2O2 did not change. However, H2O2 increased COX-2 expression, which subsequently promoted arterial vasoconstriction (73). A study demonstrated that the mitochondrial electron transport chain in PVAT has a role in modulating aortic muscle contraction. This is achieved through increased production of the superoxide anion (O_2_^−^), which is subsequently converted into H_2_O_2_. In this process, H_2_O_2_ acts as an important signaling molecule to modulate the contraction of vascular smooth muscle (74). Mitochondrial decoupling and H_2_O_2_ removal increase peripheral vasoconstriction by PVAT.

#### 3.1.6 COX-derived factors

PVAT is recognized as a source of various factors that originate from adipose tissue. These factors are generated by an enzyme called cyclooxygenase (COX) and include TXA2, prostaglandin D2, prostaglandin E2, prostaglandin F2a, prostaglandin H2, and prostaglandin I (prostacyclin) (75, 76). In particular, prostacyclin is known for its vasodilatory properties. Specifically, it interacts with receptors present on blood vessels, and has an important role in protection against endothelium dysfunction and atherosclerosis (46).

#### 3.1.7 Angiotensin 1–7

All components of the renin–angiotensin–aldosterone system (RAAS) can be found in the aortic and mesenteric PVAT, with the exception of renin (77). The RAAS components have varying effects on vascular tone. One component, Angiotensin 1–7, is known to promote vasodilation through its interaction with the endothelium (78). On the contrary, angiotensin II, which is also produced by PVAT, induces vasoconstriction (79). Angiotensin 1–7 (Ang 1–7) by affecting endothelial Ang 1–7 receptor (MAS) activates eNOS and increases the NO concentration. This increase in NO leads to blood vessel dilation through the activation of BK channels (47).

### 3.2 Anti-inflammatory effects of PVAT

#### 3.2.1 Adiponectin

Adiponectin is known for its anti-atherogenic properties (80). In human atherosclerotic plaque, the two most prominent macrophage types are inflammatory M1 macrophages and anti-inflammatory M2 macrophages (81). Adiponectin inhibits the typical pro-inflammatory activity of M1 macrophages and enhances the anti-inflammatory activity of M2 macrophages, and the expression of high levels of lipocalin potentially impedes the progression of metabolic and cardiovascular disorders by facilitating the development of an anti-inflammatory macrophage phenotype (41, 82). Macrophages can be polarized towards the M1 state by interferon-γ and TNF-α, while polarization toward the M2 state occurs through the actions of interleukin (IL)-4 and IL-13. M2 macrophages also secrete the anti-inflammatory cytokine IL-10 and reduce the production of pro-inflammatory cytokines. A study suggested that lipocalin directly regulates the phenotype of macrophages and facilitates their transition from the pro-inflammatory M1 state to the anti-inflammatory M2 state (41). Furthermore, as shown in a model of collar-induced carotid atherosclerosis, adiponectin derived from PVAT has anti-atherosclerotic properties through its capacity to initiate Akt/FOXO3-dependent autophagy in macrophages (83).

#### 3.2.2 UCP1

The mitochondrial inner membrane protein UCP1 is predominantly detected in BeAT/BAT and was originally recognized as a thermogenic protein responsible for eliminating excessive energy as heat. The gene expression patterns in the mouse thoracic PVAT are remarkably similar to the patterns in interscapular BAT (iBAT), and human coronary PVAT also shows expression of brown adipocyte-specific genes such as UCP1. Recent research has revealed that UCP1 has a protective role against vascular dysfunction and atherosclerosis by inhibiting the activation of Nod-like receptor family pyrin domain-containing 3(NLRP3) inflammatory vesicles in PVAT (84). This inhibition causes a reduction in NLRP3 inflammatory vesicles, which in turn decreases the levels of pro-inflammatory factors such as IL-1β (41). Notably, UCP1 deficiency did not alter the circulating or BAT levels of inflammatory factors. Furthermore, reintroduction of UCP1 in iBAT did not revert the increased atherosclerosis in UCP1-deficient mice, implying that the vascular regulation by UCP1 can be attributed, at least in part, to UCP1 in PVAT. Gu and colleagues conducted *ex vivo* research and showed that UCP1 in PVAT directly prevents endothelial dysfunction in intact aortic rings from mouse and porcine models (32). Through co-culture studies, they found that short-term processing of PVAT with BAM15 or co-expression with IL-1β-neutralizing antibodies improved endothelium-dependent PVAT relaxation in obese individuals. In conclusion, their study provides support for the notion that UCP1 partly exerts its vasculoprotective effects through its anti-inflammatory effects in PVAT.

#### 3.2.3 Dendritic cell depletion

Previous research has demonstrated that the depletion of adipocytes expressing CD11c mRNA has a significant effect on reducing inflammatory responses in both obese visceral adipose tissue and the general circulation (85). In a murine model of type 2 diabetes mellitus (T2DM), dendritic cells predominantly accumulated in PVAT, rather than in the vessel wall itself. The buildup of dendritic cells in PVAT was related to the overproduction of proinflammatory cytokines, which in turn led to a decrease in the ability of PVAT to enhance vasodilatory and anticontractile activity in patients with T2DM. Recent investigations further indicated that depletion of dendritic cells considerably reduced the production of TNF-α and IL-6 in adipose tissue of a mouse model of type 2 diabetes, while simultaneously reducing the generation of IL-10 (38). In conclusion, depletion of dendritic cells dramatically reduces the generation of pro-inflammatory agents in diabetic PVAT, thereby attenuating chronic inflammation.

#### 3.2.4 FGF-21

FGF-21 is a member of the fibroblast growth factor gene family that has a vital role as an endocrine regulator. Its primary functions include promotion of weight loss, regulation of insulin signaling, and control of glucose and lipid metabolism (86). FGF-21-induced glucose uptake and FGF-21 anti-inflammatory effects were shown to be mediated by separate signaling channels, and FGF-21 was further found to exhibit anti-inflammatory effects, particularly in adipocytes, that were facilitated by the fibroblast growth factor receptor substrate 2/ERK1/2 signaling pathway (48).

## 4 Relationship between PVAT and cardiovascular disease and the potential of PVAT as a therapeutic target

**Table 3** summarizes the associations of PVAT with atherosclerosis, hypertension, and heart failure. We searched for potential therapeutic targets based on various aspects of the pathogenesis of these diseases as well as the cardioprotective effects of PVAT.

**Table 3:** Associations between PVAT and cardiovascular diseases.

Cardiovascular Disease	Relationship	Reference
Atherosclerosis	Lack of PVAT augmented macrophage infiltration in the perivascular area of the aorta	(16)
	Increased production of inflammatory cytokines, which resulted in vascular inflammation and increased atherosclerotic lesions in the aortic wall	(16)
	PVAT-derived APN might be one of the anti-inflammatory adipokines able to inhibit the development of atherosclerosis	(40, 83)
	NLRP3/IL-1β pathway causes arterial inflammation and fibrosis	(87)
Hypertension	PT1R Activation in PVAT Promotes Vascular Inflammation and Endothelial Dysfunction	(88)
	RAAS, particularly AGT, is highly expressed in PVAT	(89–91)
Heart Failure	lower NO bioavailability of PVAT	(92)

### 4.1 PVAT and atherosclerosis

PVAT, which envelops blood vessels, was previously believed to be an inactive and unresponsive tissue. However, emerging evidence has strongly suggested that PVAT plays a critical role in regulating vascular function and contributes to the development of atherosclerosis (16, 31, 93–95). Atherosclerosis is a chronic and progressive metabolic disease characterized by buildup of lipids, dysfunction of the endothelium, and infiltration of inflammatory cells (96). The initial trigger for atherosclerosis is dysfunction or injury to the endothelium resulting from high shear stress, which induces adherence of inflammatory cells to the damaged endothelium and leads to cholesterol buildup within the arterial wall, facilitating the development of atherosclerosis (97, 98). These observations support the theory that atherosclerosis develops from the inside to the outside, because the adhesion of inflammatory cells to the dysfunctional endothelium triggers the accumulation of cholesterol in the artery wall (97). However, there is also evidence showing that PVAT, located in the outermost layer of the arterial wall, can contribute to the development of atherosclerosis through a different mechanism known as outside-to-inside pathogenesis (99). This outside-to-inside pathogenesis often occurs through disrupted endothelial function caused by impaired function of PVAT itself or changes in its function arising from variations in physical and chemical factors in the external environment. One study showed that thermogenic PVAT in the aorta was able to restore endothelial function in senescent mice (16). Activation of PVAT in the mice by mild cold treatment improved the endothelial function and prevented the occurrence of atherosclerosis. In contrast, mice deficient in PVAT exhibited serious atherosclerotic lesions that were not attenuated by mild cold irradiation. Furthermore, absence of PVAT led to increased infiltration of macrophages in the perivascular region of the aorta as well as increased generation of inflammatory cytokines, thereby inducing increased vascular inflammation and atherosclerotic plaque in the aortic lumen (16). Meanwhile, PVAT-induced inflammation and fibrosis may be part of the pathological process for arterial stiffness. A recent study demonstrated that secretion of mature IL-1β by macrophages is dependent on triggering the NLRP3 inflammasome, a member of the Nod-like receptor family (87). And this inflammasome is involved in an intracellular mechanism that acts through caspase-1 to mobilize the proinflammatory cytokines IL-1β and IL-18. Thus, owing to their distinctive functional and biochemical properties, it can be argued that perivascular adipocytes play an important role in the initiation of inflammation in atherosclerosis (26).

Numerous studies have demonstrated that adipokines derived from PVAT have a direct effect on the progression of atherosclerosis (100–103). A potential role for adipokines derived from subcutaneous adipocytes in atherosclerosis has also been suggested (40, 83).

Grafting of wild-type mouse thoracic PVAT to the carotid arteries of ApoE^−/−^ mice significantly decreased the plaque macrophage content, without affecting plaque size (83). In contrast, transplantation of thoracic PVAT from ApoE^−/−^ mice resulted in elevated amounts of inflammatory cytokines compared with transplantation of wild-type PVAT (104). In addition, melatonin was able to maintain the anti-contractile activity of PVAT and increase the expression of adiponectin and its receptors (105).

Another study demonstrated the anti-inflammatory effects of a cyclopentane triterpenoid called (16S,20S,24R)-12β-acetoxy-16,23-epoxy-24,25-dihydroxy-3β-(β-D-xylopyranosyloxy)-9,19-cyclolanost-22 (23)-ene (AEDC), derived from the buttercup family (Ranunculaceae). This compound showed promising results in the treatment of LPS-264.7 macrophages because it inhibited IL-1β generation and secretion. The underlying mechanism for the suppression involved SIRT3 autophagy-mediated inactivation of NLRP3 inflammatory vesicles and SIRT3-SOD2-mediated scavenging of reactive oxygen species (106). AEDC not only prevented inflammatory crosstalk between macrophages and adipocytes but also blocked the migration of macrophages to adipocytes. By mitigating macrophage accumulation, AEDC effectively alleviated adipose tissue inflammation. Therefore, there is a need to further develop AEDC as a potential drug of choice for the treatment of adipose tissue inflammation and related metabolic diseases.

### 4.2 PVAT and hypertension

Hypertension is a significant risk factor for various medical conditions, including stroke, aortic aneurysm, and coronary artery disease. It is characterized by a gradual increase in arterial blood pressure. Although contributing factors to hypertension include problems with the heart, kidneys, and nervous system, research has demonstrated that obesity also has a role in its development and progression. Existence of PVAT in obese individuals was found to reduce the contractile reaction of vascular rings. Nevertheless, this anti-contractile activity was markedly attenuated in obese individuals and obese mice (107). The pathogenesis of hypertension is complex and multifactorial, with various mechanisms by which adipose tissue could be involved in its development, particularly through variations in the secretion of adipokines. Adipocytes secrete numerous substances that affect vascular tone, and in obesity, their expression of vasodilators, such as lipocalin, NO, and H_2_S, is reduced. Adipose tissue also has its own RAAS, with angiotensinogen(Agt) being highly expressed in obese adipose tissue and potentially leading to renal dysfunction. Abnormal RAAS activation is crucial in the primary and later stages of hypertension. Elevated levels of Agt were observed in adipose tissue of rats suffering from primary hypertension and obesity, and the levels and capacities of Agt, plasma renin, and angiotensin-converting enzyme in the adipose tissue were directly correlated with obesity (89–91). These observations suggest that adipose tissue may be the main source for the RAAS in obese hypertensive patients (108). Locally, angiotensin II is derived from Agt, which is also present in PVAT. The entire vessel wall expresses four angiotensin receptors, and angiotensin type 1 receptor (AT1R) activation in PVAT enhances vascular inflammation and endothelial dysfunction (88).

Renin-angiotensin antagonists have been observed to maintain the anti-contractile function of perivascular tissues. In one study, researchers conducted *in vitro* hypoxic experiments that simulated the disinhibition of the anti-contractile function of PVAT in obese patients (109). Specifically, they contracted the tissue with increasing amounts of norepinephrine under normoxia or hypoxia and then incubated the tissue with captopril or telmisartan. Their findings showed that renin-angiotensin antagonists could effectively prevent the loss of the anti-contractile function of PVAT.

In addition, aldosterone may directly influence PVAT by promoting a proinflammatory phenotype. Angiotensin receptor blockers (ARBs) have potential as therapeutic targets by reducing the release of angiotensin II and aldosterone through the angiotensin-converting function of PVAT. ARBs also promote the generation of perivascular relaxing factors, which generate vasodilation by opening voltage-dependent K-channels on vascular ECs (110, 111). RAAS inhibition by ARBs and aldosterone inhibitors induces lower blood pressure and provides cardiovascular benefits, with effects that extend beyond the main target organs, such as the kidney and heart, to also affect PVAT (112).

SIRT3 has been identified as a regulator of glycolysis-dependent NLRP3 inflammatory vesicle activation, suggesting that SIRT3 may have potential as a therapeutic target for reducing PVAT inflammation. A recent study showed that bone marrow SIRT3 deficiency aggravated PVAT remodeling, leading to macrophage infiltration and adipose tissue dysfunction (84). Furthermore, NLRP3 deficiency protected macrophage function and prevented hypertension-induced inflammatory damage to PVAT.

### 4.3 PVAT and heart failure

Enhanced RAAS activation throughout the body, accompanied by heightened amounts of angiotensin II in the bloodstream, plays a crucial role in heart failure. Endothelial dysfunction, as a consequence of heart failure, is closely related to RAAS activation (113). A study even demonstrated a decrease in the anti-contractile effect of PVAT in the thoracic aorta of rats with heart failure (114). This reduction in the effect of PVAT may be attributed to the considerably decreased availability of NO. Existing literature indicates that decreased NO availability is a common occurrence in heart failure-induced endothelial dysfunction (92, 115). This reduced bioavailability of NO may arise through a decrease in its synthesis and/or an increase in its degradation by reactive oxygen species (92).

The above studies emphasize the potential contribution of PVAT to the pathophysiology of vascular dysfunction in heart failure and provide novel insights into the management of this disease. Thus, the use of RAAS inhibitors and the promotion of increased NO in patients with heart failure may be useful therapeutic strategies for the treatment of heart failure.

## 5 Discussion

Following the discovery of the anti-constrictive properties of PVAT in 1991, growing numbers of basic and clinical investigations have revealed important roles of PVAT in the cardiovascular system, its structural composition and cellular origins, and the release of various vasoactive molecules, thereby highlighting its essential effects on the cardiovascular system. Moreover, many studies have demonstrated its value as a possible therapeutic target from the viewpoint of its protective functions in the normal physiological state. Therefore, PVAT has potential as a candidate therapeutic target for restoring, delaying, and/or counteracting vascular dysfunction.

## Author contributions

YT: Writing – original draft. ZZ: Writing – original draft. XL:Writing – original draft. ML: Software, Writing – original draft. ZW: Software, Writing – original draft. XG: Writing – original draft. XW: Writing – original draft. YS: Writing – original draft. ZGZ: Writing – review & editing. DC: Writing – review & editing.
